# Up-to-date results of carbon-ion radiotherapy for prostate cancer

**DOI:** 10.1093/jrr/rrt177

**Published:** 2014-03

**Authors:** Takuma Nomiya, Hiroshi Tsuji, Katsuya Maruyama, Tadashi Kamada, Hirohiko Tsujii

**Affiliations:** National Institute of Radiological Sciences, Research Center Hospital for Charged Particle Therapy, 4-9-1, Anagawa, Inage-ku, Chiba 263-8555, Japan

**Keywords:** carbon-ion radiation therapy, prostate cancer, hypofractionation

## Abstract

Carbon ion radiotherapy (C-ion RT) for prostate cancer was started in 1995 using the Heavy-Ion Medical Accelerator in Chiba (HIMAC) at the National Institute of Radiological Sciences (NIRS), Japan. After preceding phase I/II dose-escalation studies of 20 fractions over 5 weeks, a phase II study was initiated in April 2000 using the treatment techniques and the recommended dose fractionations established by the phase I/II studies. This study was also successfully completed in October 2003 when the C-ion RT for solid tumors, including the prostate cancer, was approved as ‘Advanced Medicine’ by the Ministry of Health, Labor, and Welfare. Since then, more than 1400 patients have been treated with C-ion RT as of February 2012, and advancement of hypofractionation has also been achieved. In this paper, the treatment outcomes in 1144 patients who underwent the established C-ion RT between April 2000 and July 2012 were analyzed.

Out of 1144 patients, 585 patients were categorized as high-risk group, which includes patients having at least one of the following conditions: T3 clinical stage, Gleason's score of 8 or higher and PSA of 20 or higher. One hundred and ninety-seven patients who met the conditions such as clinical stage of T2a or lower, Gleason's score of 6 or lower and PSA of <20 were categorized as low-risk group. Three hundred and sixty-two patients who were not included in either high- or low-risk group were categorized as intermediate-risk group. All patients were pathologically proven to have adenocarcinoma of the prostate, and Gleason's score was determined by the chief pathologist of our study group. Written consent was obtained from all patients included in the clinical study. Patients with the following conditions were not registered in the clinical trial: having distant metastases, having synchronous primary malignancy, not histologically proven cancer, without informed consent, post-operative/post-irradiation recurrence.

All patients were treated with three field irradiations (vertical one field and horizontal opposing two fields). The prostate and proximal part of the seminal vesicle were contoured as clinical target volume (CTV), and planning target volume (PTV) was set with 5–10 mm margins around the CTV. On the way of radiotherapy, a part of irradiation field of the posterior side was cut to reduce the rectal dose. The 197 patients in the low-risk group did not undergo androgen deprivation therapy (ADT), whereas the 947 patients of intermediate- and high-risk groups underwent ADT. The patients of intermediate-risk group underwent about 6 months of neoadjuvant ADT, and the patients of high-risk group also underwent about 6 months of neoadjuvant ADT and sequential adjuvant ADT for more than 18 months. The median age of all patients was 68 years, and the median follow-up time was 48.7 months (range: 3.6–151.1 months).

The 5-year overall survival rate and biochemical relapse-free rate of the entire groups was 95.7% and 91.0%, respectively. T-stage and Gleason's score were significant prognostic factors for both the biochemical control and patient survival and initial PSA was also a predictive factor for survival. Regarding the late radiation toxicity, the incidence of rectal toxicity of grade 2 or worse was 1.1% and that of genitourinary toxicity was 6.5%. These outcomes seemed to be better than those of the past publications [ 1– 4]. In addition, the incidence of toxicity in patients treated with more hypofractionated C-ion RT of 16 fractions over 4 weeks was lower than those of 20 fraction treatment. These favorable outcomes can be thought as apparent evidence of physical and biological advantages of the hypofractionated C-ion RT.
